# Targeting to Intensify M2 Macrophage Polarization Ameliorate Podocyte Lipid Accumulation and Damage by Tea Polyphenols via Activating SIRT1 in the Aged Model Rats With DKD

**DOI:** 10.1002/fsn3.70736

**Published:** 2025-08-11

**Authors:** Shuangzhi Chen, Xi Wang, Chengyang Li, Le Cheng, Chenhui Lv, Lushan Xue, Cheng Zhang, Xuemin Li, Mingkai Li, Qinfei Guo, Yafei Zhao, Haifeng Zhao

**Affiliations:** ^1^ Nutritional and Food Science Research Institute, Department of Nutrition and Food Hygiene, School of Public Health Shanxi Medical University Taiyuan Shanxi China; ^2^ The Second Clinical Medical College of Shanxi Medical University Taiyuan China; ^3^ Center for Disease Control and Prevention in Shanxi Province Taiyuan Shanxi China; ^4^ MOE Key Laboratory of Coal Environmental Pathogenicity and Prevention Ministry of Education Taiyuan China

**Keywords:** aging, diabetic kidney disease, inflammation, lipid accumulation, macrophage polarization, tea polyphenols

## Abstract

Tea polyphenols (TP), as representative bioactive compounds of tea, exhibit anti‐inflammatory and hypolipidemic effects on aging‐associated Diabetic kidney disease (DKD), but the exact mechanism is unclear. Inflammation resulting from the dynamic imbalance of macrophage polarization and the injury of podocytes caused by lipid accumulation together drives the disease process. This study aims to explore the mechanism of TP alleviating aging with DKD via macrophage polarization and podocyte lipid accumulation. Initially, aging with DKD model rats were treated with or without TP (75, 150, 300 mg/kg once daily, ig) for 8 weeks; lipid accumulation in podocytes, inflammatory cytokines in serum and kidney, and macrophage phenotype in kidney were detected. We found silencing information regulator 1 (SIRT1), a key protein of cell senescence; its activation contributes to the transition of macrophages towards an anti‐inflammatory phenotype. (−)‐Epigallocatechin gallate (EGCG), the most important monomeric compound of TP, has been found to stably bind to SIRT1 by molecular docking experiment. Furthermore, an indirect co‐culture system of RAW264.7 and MPC5 cells was constructed to investigate the effect of EGCG on the targeted macrophage polarization, ameliorating podocyte lipid accumulation. The agonist and inhibitor of SIRT1 were used to validate through immunofluorescence analysis, Oil Red O staining, lipid‐related protein analysis, and phalloidin marking. We demonstrated that TP promotes SIRT1 activation, thereby enhancing the transformation of macrophages into the M2 phenotype, reducing renal inflammation, and ultimately alleviating podocyte lipid accumulation. Our study provides a new insight into the ways in which tea and its chemicals protect DKD in the elderly.

## Graphical abstract

Tea polyphenols (TP), as representative bioactive compounds of tea, promote SIRT1 activation, thereby enhancing the transformation of macrophages into the M2 phenotype, reducing renal inflammation, and ultimately alleviating podocyte lipid accumulation. Our study provides a new insight into the ways in which tea and its chemicals protect DKD in the elderly.

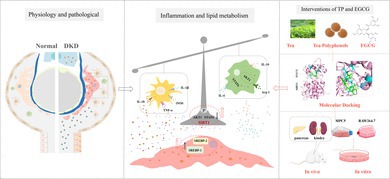

**Abbreviations**
BUN: blood urea nitrogen
CKD: chronic kidney disease
DKD: diabetic kidney disease
DM: diabetes mellitus
EGCG: (−)‐Epigallocatechin gallate
ERSD: end‐stage renal disease
FBG: fasting blood glucose
HDL: high‐density lipoprotein
IL‐1β: interleukin‐1 beta
IL‐4: interleukin 4
IL‐10: interleukin 10
IL‐18: interleukin 18
iNOS: inducible nitric oxide synthase
IRS1: insulin receptor substrate 1
LDL: low‐density lipoprotein
NAD+: nicotinamide adenine dinucleotide
PTP1B: protein tyrosine phosphatase 1B
Scr: serum creatinine
SIRT1: silent information regulator 1
SREBP: sterol regulatory element‐binding protein
STAT6: signal transducer and activator of transcription 6
T2DM: type 2 diabetes mellitus
TC: total cholesterol
TEM: transmission electron microscopy
TG: triglyceride
TNF‐α: tumor necrosis factor‐α
UACR: urinary albumin/creatinine ratio
UTP: urinary total protein

## 1 Introduction

Diabetic kidney disease (DKD), a chronic kidney disease (CKD) caused by diabetes mellitus (DM), has become the main cause of end‐stage renal disease (ESRD). In some Asian countries and the United States, the incidence of ESRD due to diabetes is highest (Johansen et al. 2023). According to the IDF ATLAS REPORTS, up to 40% of people living with diabetes develop CKD, and type 2 diabetes (T2DM) is the largest factor contributing to the burden of diabetes‐related CKD (Miyamoto and Pavkov 2023). Globally, the number of new cases of CKD due to T2DM increased from about 1.4 million in 1990 to 2.4 million in 2017, an increase of 74% (Li et al. 2021). The prevalence of DKD increases with age. A systematic review and meta‐analysis from 20 cohorts including 41,271 patients with T2DM showed a 38% increased risk of DKD with an increase in age of 5–10 years (Jiang et al. 2020). With the rapid aging of the global population, the burden of DKD is expected to increase significantly, endangering human health and seriously affecting global socioeconomic development.

As highly differentiated epithelial cells, podocyte injury is the key mechanism that drives the progression of DKD (Reidy et al. 2014). Inflammation and altered lipid metabolism play a pivotal role in podocyte injury (Luo, Chen, et al. 2024; Luo, Luo, et al. 2024). There is evidence that podocytes are abnormally sensitive to lipid accumulation, and lipid accumulation is closely related to genes that govern lipid synthesis, uptake, oxidation, lipolysis, and efflux (Fu et al. 2020; Mitrofanova et al. 2023; Qu et al. 2024). Further, it has been verified that inflammation has a feedback impact on the expression of lipid metabolism‐related genes in vivo and in vitro experiments, which can aggravate podocyte injury (Wu et al. 2021). As resident monocytic immune cells of the kidney, macrophages play an essential role in inflammation and fibrosis (Calle and Hotter 2020; Fu et al. 2019; Naaman and Bakris 2023). What is more, the activation and infiltration of macrophages can directly induce podocyte injury (Yang et al. 2019), and they have become an important marker of DKD (Naaman and Bakris 2023). Interestingly, in the microenvironment of diabetes, macrophages exhibit more M1 and less M2 phenotype, with M1 promoting an inflammatory response and M2 conversely performing more of an anti‐inflammatory effect (Cantero‐Navarro et al. 2021; Lin et al. 2024). Recently, ameliorating macrophage polarization imbalance and crosstalk with kidney cells has been widely recognized as a promising macrophage‐related therapeutic strategy in DKD (Li et al. 2022).

Tea, as a popular beverage worldwide, has a health‐promoting effect on the prevention and treatment of DM. A cohort study of approximately 500,000 adults in China with a follow‐up of 11.1 years (Nie et al. 2021), and an EPIC‐InterAct case cohort study for a total of 340,234 participants in 8 European countries with 3.99 million person‐years of follow‐up (InterAct et al. 2012), both suggest that daily tea consumption reduces the risk of developing T2DM. In addition, a Mendelian randomized study implied that drinking tea per day has a protective effect on CKD G3‐G5 [OR = 0.803; *p* = 0.004] (Zhang et al. 2022). TP, the main active substance in tea, exists with potential health benefits such as anti‐inflammatory, anti‐diabetic activity, and hypoglycemic effects, etc. (Luo, Luo, et al. 2024). It has also been found that EGCG (the most important monomeric compound in TP) ameliorates DKD by inhibiting endoplasmic reticulum stress‐associated NLRP3‐mediated inflammation (Zhang et al. 2024).

Silent information regulator 1 (SIRT1), a member of the NAD + ‐dependent class III deacetylase family, has become a new target for the prevention and treatment of DKD, seemingly regulated by polyphenolic compounds (Jin et al. 2024). Previous studies have shown that SIRT1 is not only an important regulator of lipid metabolism (Shen et al. 2024; Wu et al. 2022) but also is known for its anti‐inflammatory properties (Wu et al. 2022; Yang et al. 2022). Specific knockout of SIRT1 was found to affect the transformation of macrophage phenotypes in mice of an abdominal aortic aneurysm model (Zhang et al. 2018). However, the exact mechanism of the alteration of macrophage phenotype is not entirely clear. Based on the targeting strategy for SIRT1‐mediated macrophage polarization, a series of experiments are employed to investigate the specific mechanism by which targeting macrophage polarization ameliorates lipid accumulation in the kidney. In summary, in this study, we aimed to provide novel insights into the mechanism by which TP ameliorates aging with DKD.

## 2 Materials and Methods

### 2.1 Reagents

TP (from green tea polyphenols, purity ≥ 98%) was purchased from Xi'an Lincao Bioengineering Co. Ltd. (HTP‐98‐211,215, Xi'an, China). EGCG was provided by MedChemExpress (purity ≥ 99%, HY‐13653, USA). Streptozocin (STZ) is produced and detected ≥ 98% (HPLC) by Sigma‐Aldrich (S0130, USA). D‐galactose was from Dalian Bogreen Biotechnology Co. Ltd. (purity ≥ 98%, MB1853, Dalian, China). Palmitic acid BSA high lipid cell supplement (PA) was obtained from Xi'an Kunchuang Biotechnology (KC1003, Xi'an, China).

### 2.2 Animals and Experimental Design

**FIGURE 1:**
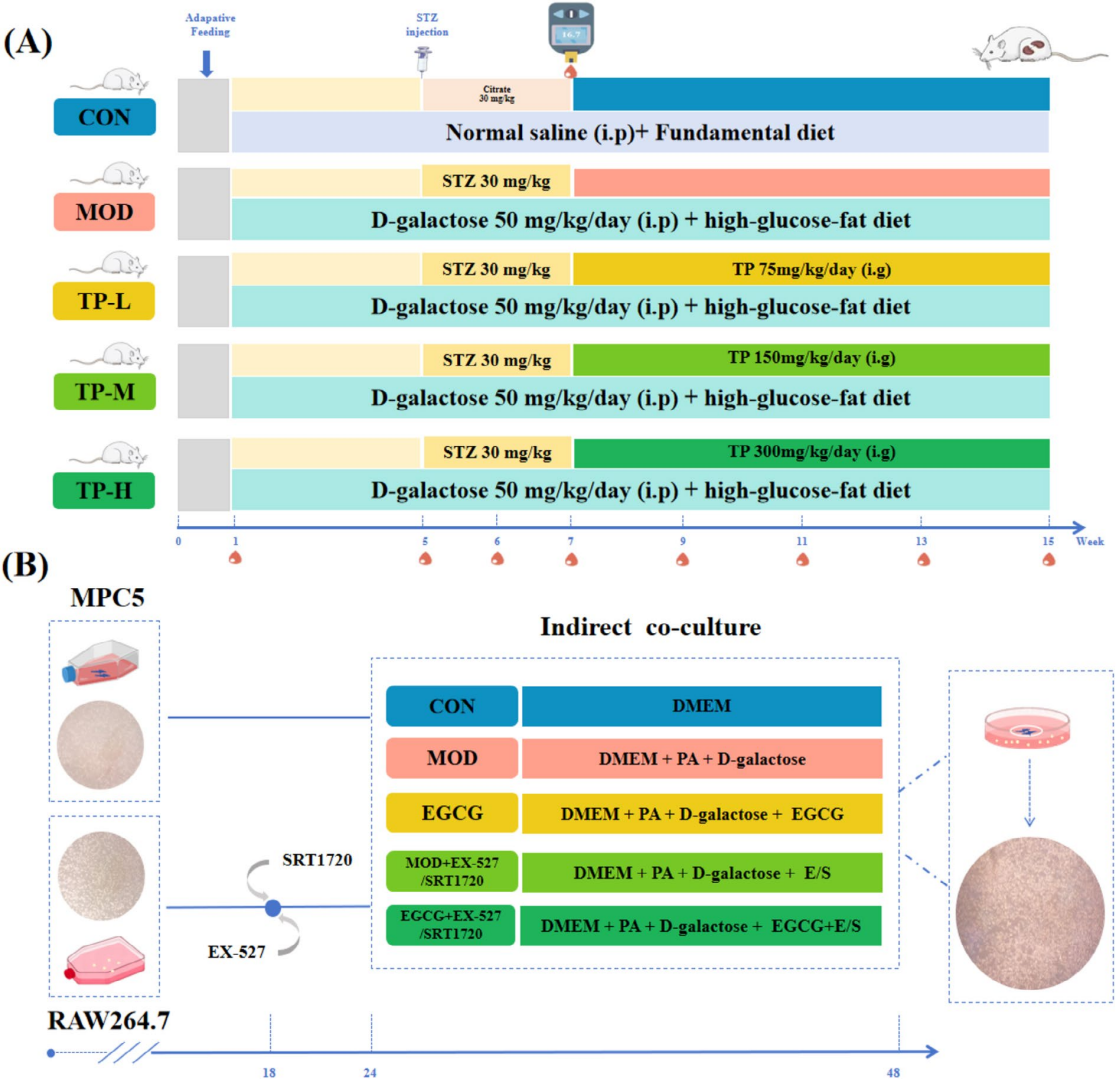
The design of animal and cell experiments. (A) The treatment of rats in each group. Red blood drops indicate that FBG was detected at the corresponding time point. High‐glucose and high‐fat diets consist of 66.5% standard chow diet, 20% lard, 10% sucrose, 2.5% cholesterol, and 1% sodium cholate. (B) The treatment of cells in each group.

Healthy adult male Sprague–Dawley (SD) rats (300 ± 20 g, *n* = 50) were purchased from the China National Institutes for Food and Drug Control (Beijing, China), with the permission number SCXK (Jing) 2022‐0002. The laboratory conditions were maintained at a suitable temperature and humidity. All the animals were housed under standardized conditions on a 12‐h light/dark cycle, with unrestricted access to food and water. After adaptive feeding for 1 week, they were randomly divided into the control group (CON), the aging with DKD model group (MOD), the TP 75 mg/kg group (TP‐L), the TP 150 mg/kg group (TP‐M), and the TP 300 mg/kg group (TP‐H) (*n* = 10, respectively) according to the body weight. The treatment of rats in each group is shown in Figure 1A. Fasting blood glucose (FBG), body weight, food and water intake, and urine volume were recorded during the experiments. All animal experiments comply with the ARRIVE guidelines and are approved by the Institutional Animal Care and Use Committee of Shanxi Cancer Hospital (Issue No. 2022002).

### 2.3 Cell Culture

RAW264.7 cells were purchased from Pricella (CL‐0190, China) and MPC5 were provided by Abcell (AC427, China). Both cells were cultured in DMEM (Servicebio, Wuhan, China) supplemented with 10% fetal bovine serum (FBS; Every green, Zhejiang, China) and 1% penicillin/streptomycin (Solarbio, Beijing, China), and placed at 37°C in a humidified 5% CO_2_ incubator for 24 h. MPC5 cells were seeded at a density of 1 × 10^6^6^/ml in a Petri dish containing coverslips. Macrophages were seeded at a density of 1 × 10^6^6^/ml in six‐well plates containing three paraffin spots with a diameter and height of approximately 1 mm, and treated with EX‐527 (MedChemExpress, HY‐15452, USA) and SRT1720 (MedMol, S80004, China) 6 h in advance. The coverslips containing MPC5 cells were inverted into pretreated RAW264.7 cells to construct an indirect coculture system. Subsequently, the cells were randomly divided into CON group, MOD group, EGCG group, EX‐527/SRT1720 + MOD group, and EX‐527/SRT1720 + EGCG group. The treatment of cells in each group is shown in Figure 1B.

### 2.4 FBG Analysis

The FBG levels of rats were measured by the electrochemical method. The number of electrons produced by the enzyme's reaction with glucose was read by a current counting facility and converted into glucose concentration.

### 2.5 SA‐β‐Gal Staining Analysis

Kidney frozen tissue sections (4 μm thick) and treated cell samples were stained with SA‐β‐gal working solution (G1073; Servicebio, Wuhan, China) and incubated at a constant temperature of 37°C for 24 h. Then, the samples were observed by a light microscope (Olympus, Japan). The results were expressed as the percentage of SA‐β‐gal‐positive cells.

### 2.6 Serum, Urine Biochemistry, and Inflammatory Factor Analysis

The levels of Scr, BUN, HDL, LDL, TC, and TG in serum and the concentrations of urinary albumin and urinary creatinine were measured with their respective corresponding rat kits produced by Nanjing Jiancheng Bioengineering Institute, China. The levels of proinflammatory cytokines (TNF‐α, IL‐1β, and IL‐18) and anti‐inflammatory cytokines (IL‐4 and IL‐10) in serum and cell culture supernatant concentrations were determined using ELISA kits (Mlbio, China). These indicators were analyzed following the manufacturer's protocols.

### 2.7 Hematoxylin and Eosin (HE) and Masson Staining

Kidney and pancreatic tissues were fixed in 4% paraformaldehyde for 48 h, dehydrated, embedded in paraffin, and sectioned into 4 μm slices. Kidney tissue sections were prepared for HE and Masson staining, and the pancreatic tissue sections were only prepared for HE staining. Subsequently, images were obtained with an optical microscope (Olympus, Japan) at 100× and 200× magnifications.

### 2.8 Oil Red‐O Staining

Kidney frozen tissue sections (4 μm thick) and treated cell samples were fixed in 4% paraformaldehyde, soaked in 1% Oil Red O for 30 min, and stained in 60% Harris modified hematoxylin solution for 1 min. Images were obtained with an optical microscope (Olympus, Japan) and analyzed via ImageJ software.

### 2.9 Transmission Electron Microscopy (TEM)

The kidney tissue cut into 1 mm^3^ pieces was fixed with 2.5% glutaraldehyde and 1% osmium tetroxide, washed with PBS, and dehydrated in a graded series of ethanol solutions (30%, 50%, 70%, 80%, 90% and 100%) for 15–20 min. It was then transferred to acetone and incubated for 20 min, placed in copper mesh to make ultrathin sections (70–80 nm), double‐stained with uranyl acetate and lead citrate, and finally observed with transmission electron microscopy (Hitachi, Tokyo, JEOL, Tokyo, Japan).

### 2.10 Immunofluorescence

Kidney frozen sections (4 μm thick) were fixed with 4% paraformaldehyde for 15 min and permeabilized with 0.2% Triton X‐100 for 10 min. After blocking with goat serum for 30 min at 37°C, sections were incubated with iNOS (1:2000, GB11119), Arg‐1 (1:2000, GB11285), SYNPO (1:2000, GB151379), and adipophilin (1:500, GB115593) in PBS at 4°C overnight. Subsequently, incubation was performed with the respective corresponding secondary antibody: donkey anti‐rabbit IgG (CY3, red, 1:100; GB21403), goat anti‐mouse IgG (Alexa Fluor 488, green, 1:200; GB25301), goat anti‐mouse IgG (Alexa Fluor 488, green, 1:200; GB25301), and donkey anti‐rabbit IgG (CY3, red, 1:100; GB21403). Stained samples were visualized by fluorescence microscopy, and the results were analyzed using ImageJ.

Macrophages were grown and stimulated in 24‐well chamber slides and fixed with 4% paraformaldehyde for 15 min. We used Triton X‐100 to perforate the podocyte membrane. Then, the samples were incubated with iNOS (1:100, GB11119) and Arg‐1 (1:200, GB11285) primary antibodies overnight at 4°C. After washing, we incubated it with the secondary antibody: donkey anti‐rabbit IgG (CY3, red, 1:100; GB21403) and goat anti‐rabbit IgG (Alexa Fluor 488, green, 1:200; GB25303) for 1 h, protected from light. These antibodies were purchased from Servicebio (Wuhan, China). Images were acquired with a fluorescence microscope and evaluated by ImageJ software.

### 2.11 Molecular Docking

The interaction between EGCG and SIRT1 protein was investigated by molecular docking simulation. The molecular structure of EGCG is derived from the PubChem compound. The crystal structure of SIRT1 protein was obtained from the Uniprot database. AutoDock Vina software is used for hydrogenation, charge assignment, atom designation, and molecular docking. Data visualization of molecular docking was performed using PyMOL 2.3.2.

### 2.12 Phalloidin Staining

After finishing co‐culture with macrophages, podocytes are fixed with 4% paraformaldehyde for 10 min and permeabilized with 0.5% Triton X‐100. Then, the samples are incubated in phalloidin stain for 30 min. Images are captured using a fluorescence microscope.

### 2.13 Western Blot Analysis

The total protein of renal cortex or cultured cells was extracted with RIPA lysate and its concentration was determined according to the instructions of the BCA protein assay kit. Isometric amounts of protein were separated with SDS‐PAGE and transferred to a polyvinylidene difluoride (PVDF) membranes (IPVH00010; Millipore Corp, USA). After blocking for 2 h with 5% skim milk at room temperature, the membrane was incubated overnight at 4°C with the primary antibody. Then, treat the membrane with the corresponding secondary antibody for 1 h. The antibodies of P‐IRS1‐S307, IL‐18, IL‐10, SIRT1, and STAT6 were purchased from ABclonal (Wuhan, China); the antibodies of AKT1, P‐AKT1‐S473, and P‐STAT6‐Thy641 were bought from Affinity Biosciences (USA); the antibodies of P21, Arg‐1, TNF‐α and GAPDH were purchased from Servicebio (Wuhan, China); the antibodies of IRS1, iNOS, IL‐1β and SREBP‐2 were bought from Boster (USA); the antibodies of P53, IL‐4, and podocine were bought from Proteintech (Wuhan, China); and the antibodies of SREBP‐1 and Nerphrin were respectively purchased from HUABIO (Hangzhou, China) and Bioss (Beijing, China). These antibodies were diluted at 1:1000 except for P53, which was diluted at 1:5000. Finally, ECL (BMU101; Abbkine, Wuhan, China) was used to show protein bands. Image J software was used to quantify protein expression.

### 2.14 Statistical Analysis

Statistical data were analyzed by One‐way ANOVA. All data were expressed as mean ± SD. The histogram of the data analysis was generated by GraphPad Prism 10.0. Statistical significance was defined as *p* < 0.05.

## 3 Results

### 3.1 Effects of TP on the Typical Symptoms, IR, and Senescence in the Aged T2DM Rats

**FIGURE 2:**
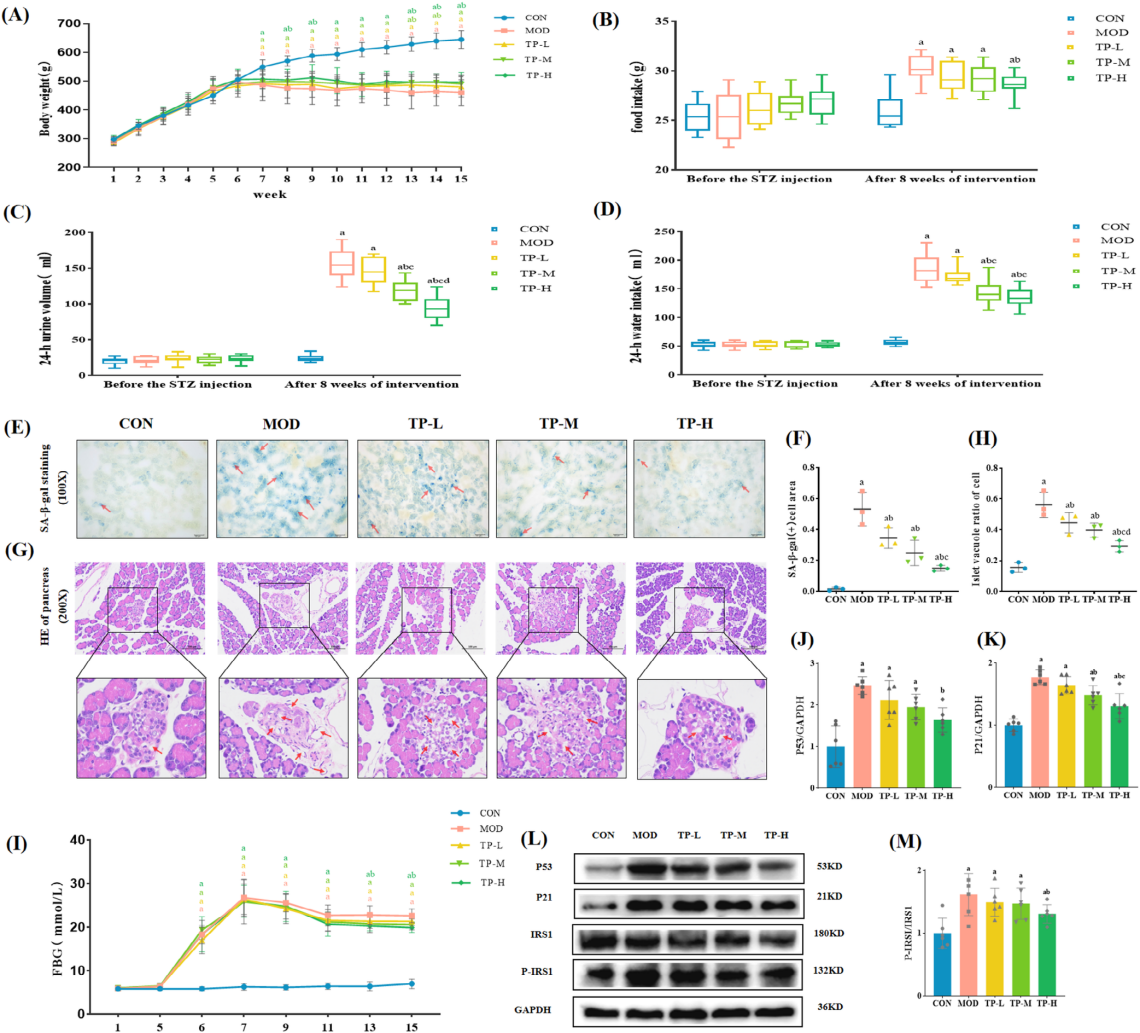
Effects of TP on the typical symptoms, IR, and senescence in the aged T2DM model rats. (A) Body weight (*n* = 10). (B) Food intake (*n* = 10). (C) 24 h urine volume (*n* = 10). (D) 24 h water intake (*n* = 10). (E, F) Representative SA‐β‐gal staining images in kidney tissues of rats and the area of SA‐β‐gal staining positive cells (100×, *n* = 3). (G, H) Representative HE images and quantitative analysis of islet vacuoles ratio to cells (200×, *n* = 3). (I) FBG (*n* = 10). (J–M) Representative WB images and quantification of the expression of P21, P53, and P‐IRS1 (*n* = 6). Compared with the CON group, ^a^ *p* < 0.05; compared with the MOD group, ^b^ *p* < 0.05; compared with the TP‐L group, ^c^ *p* < 0.05; compared with the TP‐M group, ^d^ *p* < 0.05.

Before STZ injection, there were no differences in body weight, food intake, 24‐h urine volume, 24‐h water intake, and FBG among the rats in each group (Figure 2A–DI). After STZ injection for 2 weeks, the FBG of the rats was greater than 16.7 mmol/L (this value was defined as the success of rat diabetes model) (Figure 2K). After the experiment, compared with the CON group, the MOD group showed an increased in FBG, food intake, 24‐h urine volume, and 24‐h water intake, and a decreased in body weight (Figure 2A–D,I). Additionally, HE staining of the pancreas showed irregular morphology and blurred boundaries; the islet cells presented diffuse distribution and severe vacuolation degeneration in the MOD group, which resulted in the islet vacuole ratio of cells being significantly increased compared with the CON group (Figure 2G,H). Moreover, the expression level of P‐IRS1‐S307 protein in kidney tissues in the MOD group was significantly higher than that in the CON group (Figure 2L,M). These results showed that the MOD group rats were in a state of insulin resistance (IR). Compared with the CON group, the area of SA‐β‐gal‐positive cells and the expression levels of P53 and P21 proteins in the MOD group were significantly increased, which proved that daily injection of D‐gal was effective in inducing senescence (Figure 2E,F,J–L). Daily gavage of 75, 150, or 300 mg/kg TP had a protective effect on the aged T2DM model rats constructed by STZ combined with D‐gal; furthermore, intragastric administration of different doses of TP improved insulin resistance in different groups of rats to varying degrees.

### 3.2 TP Alleviated Renal Damage in the Aged T2DM Rats

**FIGURE 3:**
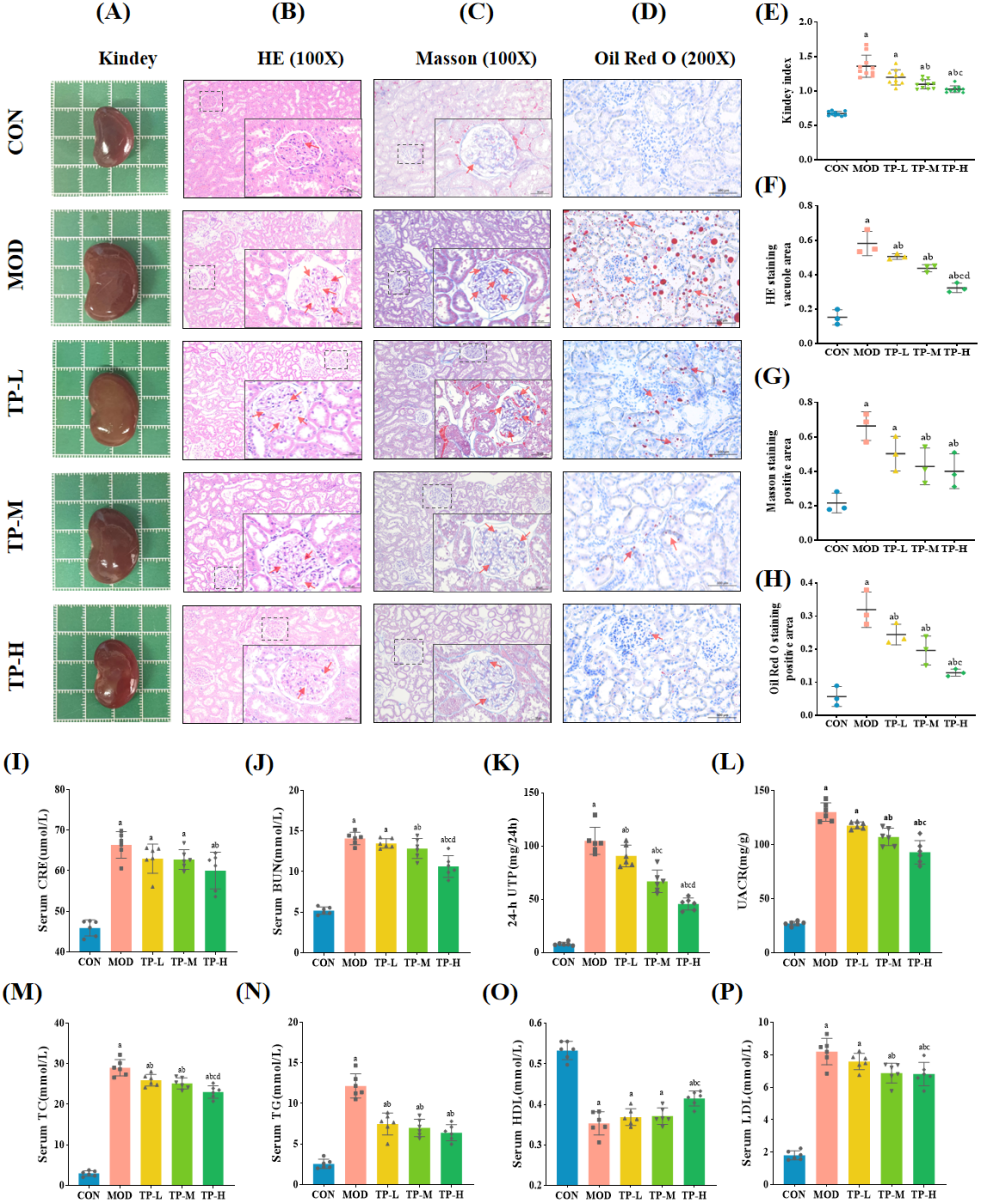
TP alleviated renal damage in the aged T2DM model rats. (A, E) Representative kidney images of rats and the kidney index (*n* = 3). (B, F) Representative H&E images and quantitative analysis of vacuole area (100×, *n* = 3). (C, G) Representative Masson images and quantitative analysis of positive area (100×, *n* = 3). (D, H) Representative Oil Red O images and quantitative analysis of positive area (200×, *n* = 3). (I, J) Levels of CRE, BUN in serum (*n* = 6). (K, L) Levels of 24‐h UTP and UACR (*n* = 6). (M–P) Levels of TC, TG, HDL, LDL in serum (*n* = 6). Compared with the CON group, ^a^ *p* < 0.05; compared with the MOD group, ^b^ *p* < 0.05; compared with the TP‐L group, ^c^ *p* < 0.05; compared with the TP‐M group, ^d^ *p* < 0.05.

Clinically, the levels of Scr, BUN, 24‐h UTP, and UACR are the parameters reflecting renal function, while the levels of TC, TG, HDL, and LDL are the indicators of lipid metabolism. As shown in Figure 3I–L, the serum levels of CRE and BUN, 24‐h UTP, and UACR in the MOD group were significantly higher than those in the CON group (*p* < 0.05). Interestingly, 8 weeks of continuous differential‐dose TP intervention reversed the damage, and the high‐dose group showed better improvement effects. Figure 3M–P showed that the levels of TC, TG, and LDL in the MOD group were significantly higher than those in the CON group, while the levels of HDL were significantly lower than those in the CON group (*p* < 0.05). Compared with the MOD group, the levels of TC, TG, and LDL decreased, and the HDL level increased in each TP intervention group.

In order to more intuitively reflect the pathological changes of the kidney, we also performed HE, Masson, and Oil Red O staining; meanwhile, the appearance of the kidney was observed, and the renal index was calculated. In Figure 3A,E, we could see that the kidney of the MOD group was significantly swollen and hypertrophied, and the renal index was significantly higher than that of the CON group (*p* < 0.05). HE staining showed that the rats in the MOD group have some significant pathological changes, such as irregular glomerular shape and obvious vacuolar degeneration compared with the CON group (Figure 3B,F). At the same time, Figure 3C,G suggested that the Masson staining of the MOD group also showed obvious collagen fiber deposition, and quantitative analysis showed that the ratio of positive area increased (*p* < 0.05). In addition, the results of Oil Red O staining indicated that there was obvious lipid accumulation in the kidneys of the MOD group (Figure 3D,H). After intervention, the above pathological injuries in the TP‐L, TP‐M, and TP‐H groups were alleviated.

### 3.3 TP Improved Podocyte Lipid Accumulation and Injury in the Aging With DKD Model Rats

**FIGURE 4:**
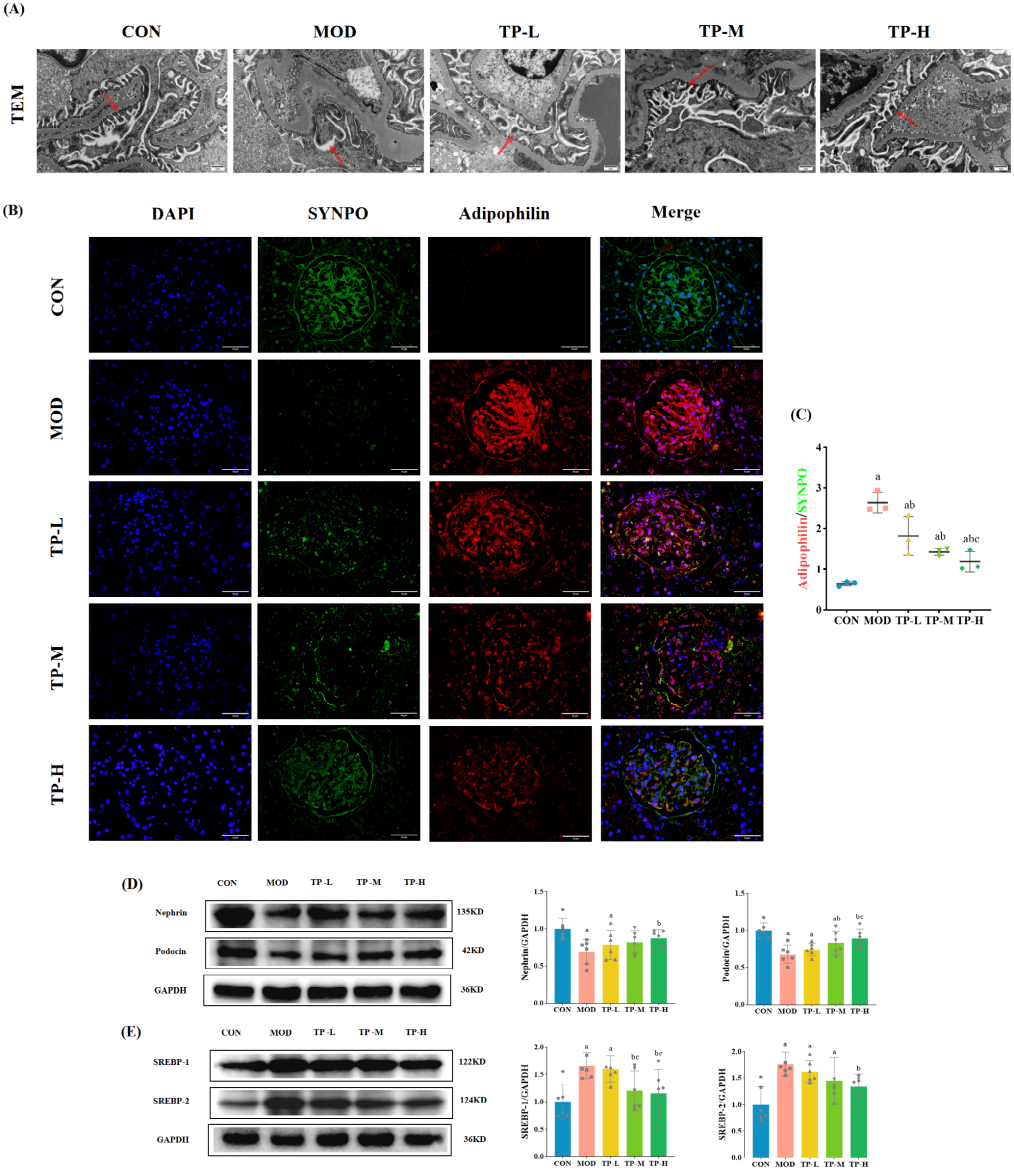
TP improved podocyte lipid accumulation and injury in the aging with DKD model rats. (A) Representative TEM images of podocytes in kidney tissues of rats. 20,000×, *n* = 3. (B) Representative immunofluorescence colocalization images of SYNPO (green fluorescence) and Adipophilin (red fluorescence). 400×, *n* = 3. (C) The analysis of mean fluorescence density ratio of Adipophilin to SYNPO assay from each group of rats. (D) Representative WB images and quantification of the expression of Nephrin and Podocin. *n* = 6. (E) Representative WB images and quantification of the expression of SREBP‐1 and SREBP‐2. *n* = 6. Compared with the CON group, ^a^ *p* < 0.05; compared with the MOD group, ^b^ *p* < 0.05; compared with the TP‐L group, ^c^ *p* < 0.05; compared with the TP‐M group, ^d^ *p* < 0.05.

To further evaluate renal damage, the ultrastructure of glomeruli was observed by transmission electron microscopy (TEM). As shown in Figure 4A, the glomerular basement membrane of the CON group had a clear morphology and normal structure; the podocytes were regular in shape and neatly arranged. While the basement membrane in the MOD group was widely thickened, the podocytes were enlarged, the number of podocytes was reduced, and the podocytes were disordered, fused, and disappeared. The TP‐L, TP‐M, and TP‐H groups of rats had a certain degree of improvement in the glomerular basement membrane, foot process fusion, and podocyte number and arrangement. In addition, the expression levels of podocyte‐specific proteins—nephrin and podocin were—quantitatively analyzed. Compared with the CON group, these protein expression levels in the MOD group were significantly reduced (*p* < 0.05), while the expression of nephrin and podocin in the TP‐L, TP‐M, and TP‐H groups increased to varying degrees relative to the MOD group (Figure 4D).

Based on our observations of the Oil Red O results, we evaluated the effect of different doses of TP intervention on podocyte lipid metabolism. Co‐staining with Adipophilin (a lipid droplet marker) and SYNPO revealed that the MOD group had a lower number of podocytes and more severe lipid accumulation, while the CON group had more podocytes without lipid accumulation (Figure 4B). As can be seen in Figure 4C, this situation has been improved in a stepwise manner with the further increase of the intervention dose of TP. Podocyte lipid accumulation has been reported to be closely related to fatty acid and cholesterol synthesis. Therefore, we measured the expression levels of SREBP‐1 and SREBP‐2 (Figure 4E). Compared with the CON group, the expression levels of SREBP‐1 and SREBP‐2 in the MOD group were significantly increased (*p* < 0.05). Compared with the MOD group, the expression levels of SREBP‐1 and SREBP‐2 in the TP‐L, TP‐M, and TP‐H groups were decreased, and the TP‐H group showed significant differences (*p* < 0.05).

### 3.4 TP Corrected Inflammation Levels in the Aging With DKD Model Rats

**FIGURE 5:**
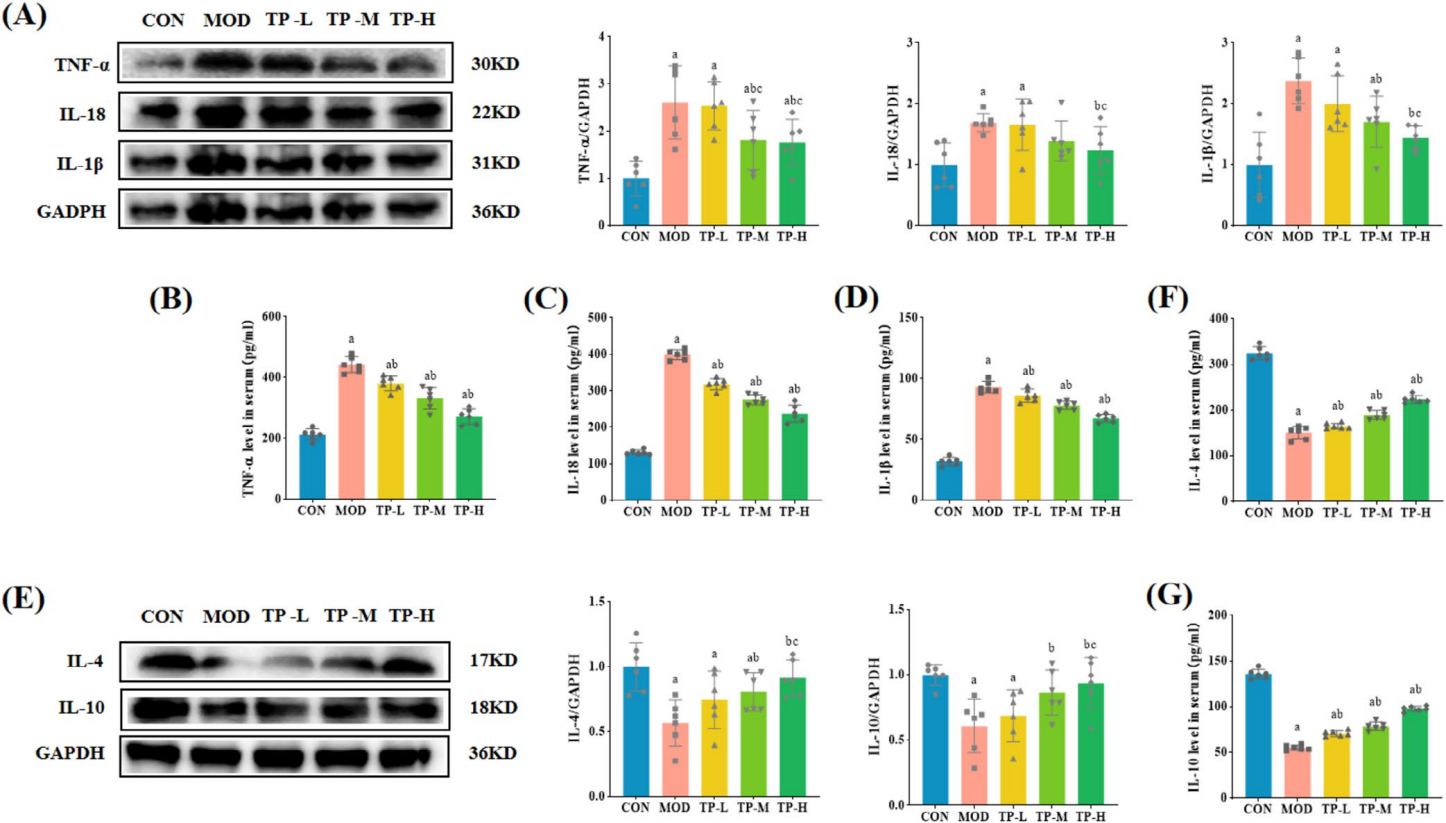
TP corrected inflammation levels in the aging with DKD model rats. (A) Representative WB images and quantification of the expression of TNF‐α, IL‐1β, and IL‐18 (*n* = 6). (B–D) Levels of TNF‐α, IL‐18, and IL‐1β in serum (*n* = 6). (E) Representative WB images and quantification of the expression of IL‐4 and IL‐10 (*n* = 6). (F, G) Levels of IL‐4 and IL‐10 in serum (*n* = 6). Compared with the CON group, ^a^ *p* < 0.05; compared with the MOD group, ^b^ *p* < 0.05; compared with the TP‐L group, ^c^ *p* < 0.05; compared with the TP‐M group, ^d^ *p* < 0.05.

An important hallmark of DKD has been reported to be aseptic inflammation. Therefore, we measured the levels in serum and protein expressions in the kidney of the pro‐inflammatory factors TNF‐α, IL‐1β, and IL‐18, and the anti‐inflammatory factors IL‐4 and IL‐10. ELISA and WB results (Figure 5A–D) showed that the levels of TNF‐α, IL‐1β, and IL‐18 were significantly higher in the MOD group compared to the CON group (*p* < 0.05). After the intervention of TP, the level of inflammation was improved. In contrast, Figure 5E–G showed that serum concentrations and protein expression levels of IL‐4 and IL‐10 were significantly lower in the MOD group compared to the CON group (*p* < 0.05). The serum concentrations of IL‐4 and IL‐10 in the three dose TP treatment groups increased significantly compared with the MOD group (*p* < 0.05); the protein expression levels of IL‐4 and IL‐10 in the TP‐M and TP‐H groups were also significantly increased (*p* < 0.05), but there was no significant difference in the TP‐L group.

### 3.5 TP Inhibited Macrophage M1‐Like Transitions in Kidney of the Aging Rats With DKD Model Rats

**FIGURE 6:**
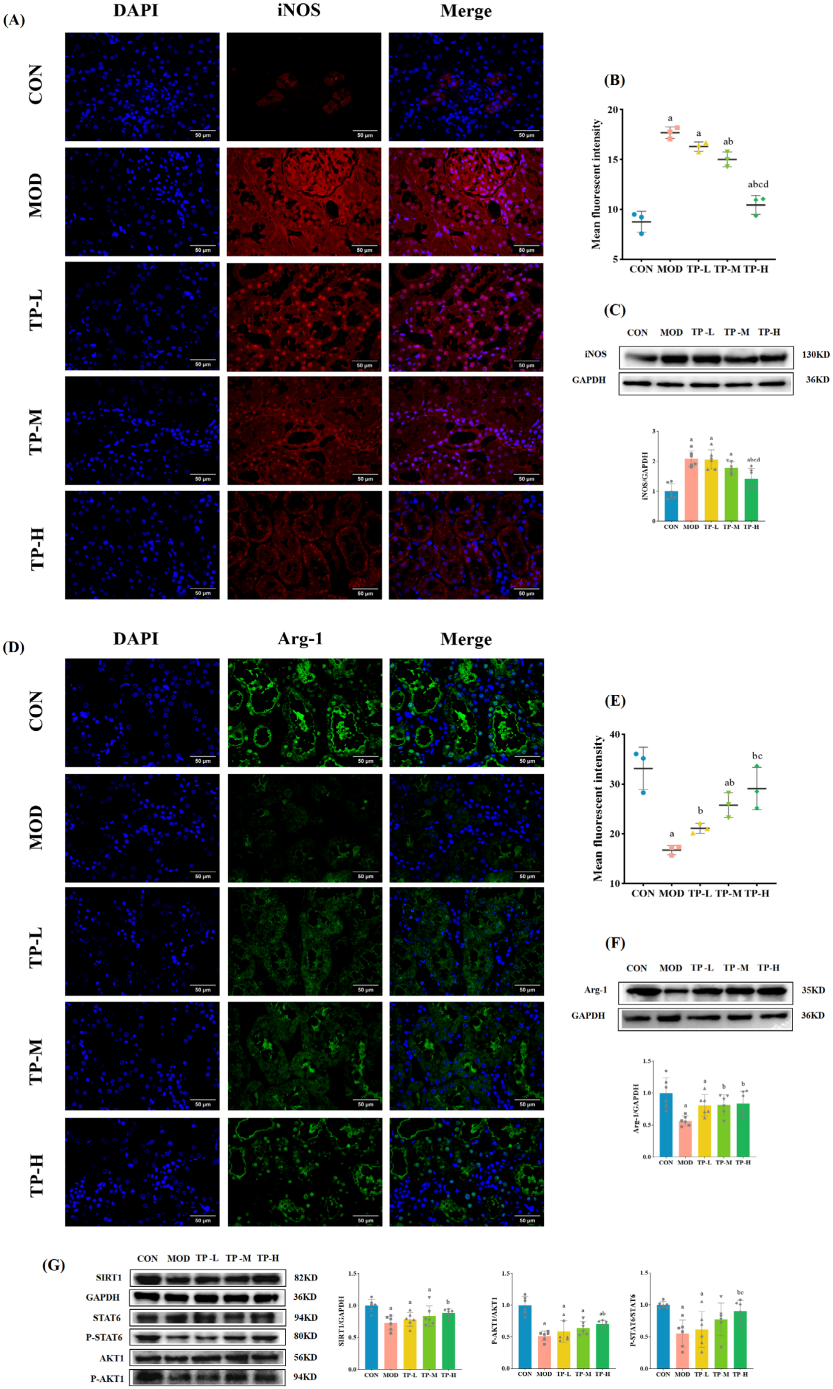
TP inhibited macrophage M1‐like transitions in kidney of the aging with DKD model rats. (A) Representative iNOS immunofluorescence images (Red fluorescence). 400 ×, *n* = 3. (B) The mean fluorescent intensity in iNOS assay of each group of rats. (C) Representative WB images and quantification of the expression of iNOS. *n* = 6. (D) Representative Arg‐1 immunofluorescence images (Green fluorescence). 400×, *n* = 3. (E) The mean fluorescent intensity in Arg‐1 assay of each group of rats. (F) Representative WB images and quantification of the expression of Arg‐1. *n* = 6. (G) Representative WB images and quantification of the expression of SIRT1, P‐STAT6 and P‐AKT1. *n* = 6. Compared with the CON group, ^a^ *p* < 0.05; compared with the MOD group, ^b^ *p* < 0.05; compared with the TP‐L group, ^c^ *p* < 0.05; compared with the TP‐M group, ^d^ *p* < 0.05.

To further explore the role of macrophage phenotype in inflammation, we performed immunofluorescence staining for their M1 and M2‐like specific proteins, which are iNOS and Arg‐1. Fig. 6A and B showed that the immunofluorescence intensity of iNOS in the MOD group was significantly higher than that of the CON group (*p* < 0.05). Compared with the MOD group, the mean fluorescence density of iNOS in the TP‐M and TP‐H groups was significantly reduced (*p* < 0.05), but there was no difference between the TP‐L and MOD groups. Furthermore, the protein expression level of iNOS also further confirmed this result (Figure 6C). The results of immunofluorescence of the M2‐like macrophage‐specific protein Arg‐1 (Figure 6D,E) showed that the mean immunofluorescence intensity of the MOD group was significantly lower than that of the CON group (*p* < 0.05). After the intervention, the mean fluorescence density of Arg‐1 in each TP treatment group increased (*p* < 0.05). Moreover, the WB results (Figure 6F) showed that the protein expression level of Arg‐1 in the MOD group decreased compared with the CON group (*p* < 0.05). Compared with the MOD group, the protein expression levels of TP‐M and TP‐H increased (*p* < 0.05), but there was no difference between TP‐L and the MOD group.

Furthermore, to explore the mechanisms influencing phenotypic shifts in macrophages, we tested several key proteins. We found that the phosphorylation of AKT1 and STAT6, as well as the protein expression levels of SIRT1 in the TP‐H group were higher than those in the MOD group (*p* < 0.05), but in the TP‐L and TP‐M groups, there was no significant difference (Figure 6G).

### 3.6 EGCG Relieved Senescence, IR, and Lipid Accumulation in the MPC5 Model Cells

**FIGURE 7:**
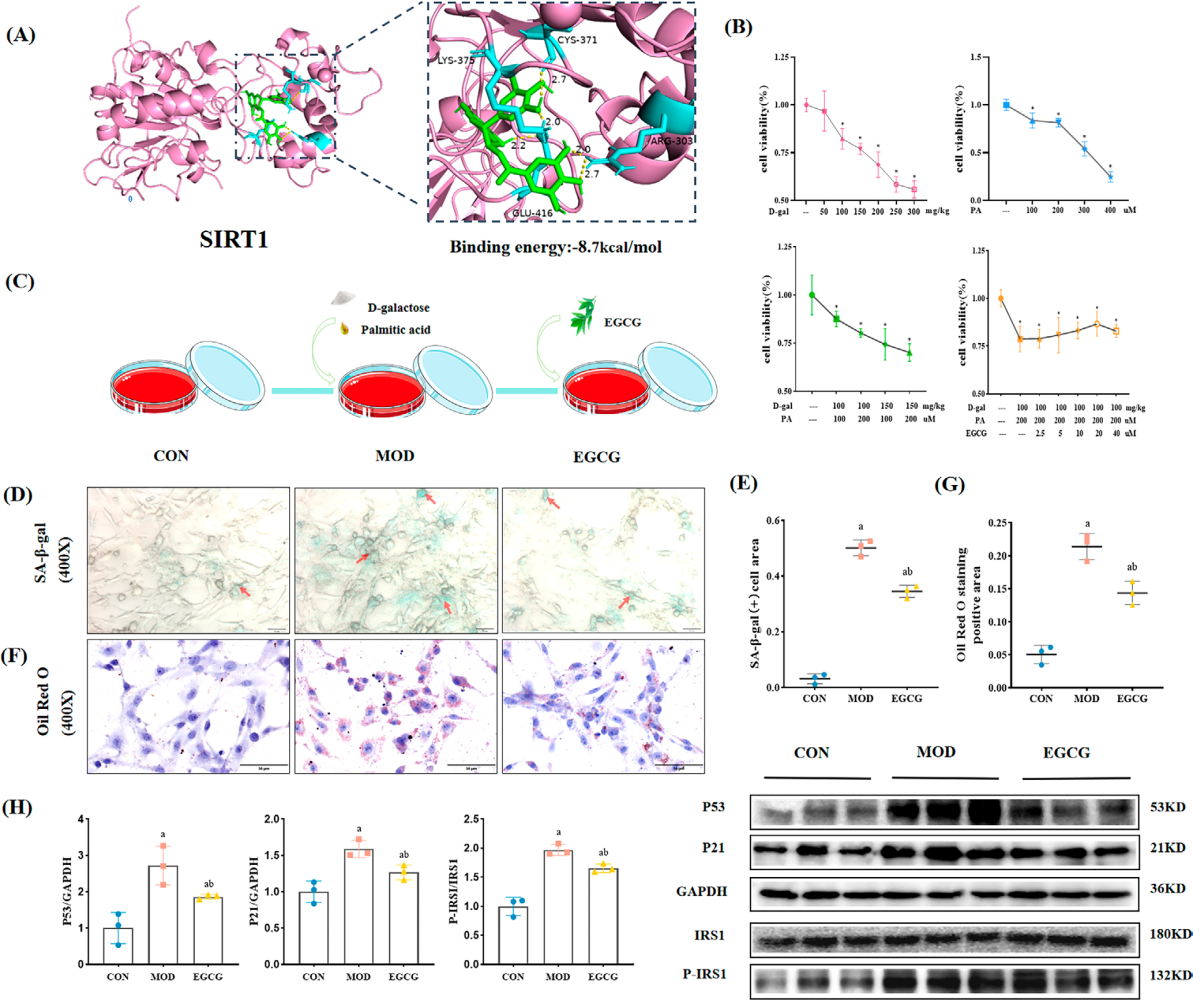
EGCG relieved senescence, IR, and lipid accumulation in the MPC5 model cells. (A) Molecular docking predicts the interaction of EGCG with SIRT1. (B) Determination of the optimal concentration of D‐galactose, PA, D‐galactose combined with PA and EGCG. *n* = 6. (C) The treatment of cell modeling in each group. (D, E) Representative SA‐β‐gal staining images of MPC5 cells and the ratio of SA‐β‐gal‐positive cells. 400×, *n* = 3. (F, G) Representative Oil Red O images of MPC5 cells and quantitative analysis of positive area. 400×, *n* = 3. (H) Representative WB images and quantification of the level of P21, P53, and P‐IRS1. *n* = 3. Compared with the CON group, ^a^ *p* < 0.05; compared with the MOD group, ^b^ *p* < 0.05. * symbol that the cell viability (%) of different group compared with the group of without any reagents, *p < 0.05.

EGCG is the main active substance of tea polyphenols, and we use it as an intervention for subsequent cells. First, to detect the interaction of EGCG with SIRT1, which is a key protein in macrophage polarization, we performed molecular docking. Figure 7A shows that EGCG has the potential binding capacity to SIRT1, and the screening criteria for binding energy less than −5.0 kcal/mol were used. The results support that EGCG can modulate macrophage polarization through SIRT1.

Next, we validated the association between macrophage polarization and podocyte lipid accumulation in vitro. CCK8 results (Figure 7B) showed that D‐galactose 100 mg/kg and Palmitic acid 200 μM significantly reduced cell viability, and the cell viability rate of the combined treatment was greater than 80% (*p* < 0.05). 20 μM EGCG treatment had the highest cell viability compared to the MOD group. Therefore, the above concentrations were used for simulating an in vitro cellular environment similar to that in vivo in animals (Figure 7C). To determine if the model can well simulate aging and IR, SA‐β‐gal staining, Oil Red O staining, and protein expression levels of P53, P21, P‐IRS1, and IRS1 were detected (Figure 7D–H). The results showed that the MOD group had a significant increase in the ratio of SA‐β‐gal positive cells, the area of Oil Red O staining, and the expression levels of P53, P21, and P‐IRS1 compared to the CON group. After the treatment of EGCG, all these indicators decreased compared to the MOD group, which suggests that EGCG can relieve senescence, IR, and lipid accumulation in the model cells.

### 3.7 SIRT1 Inhibitor EX‐527 Increased M1‐Like Macrophage and Lipid Accumulation by EGCG in the Model Cells

**FIGURE 8:**
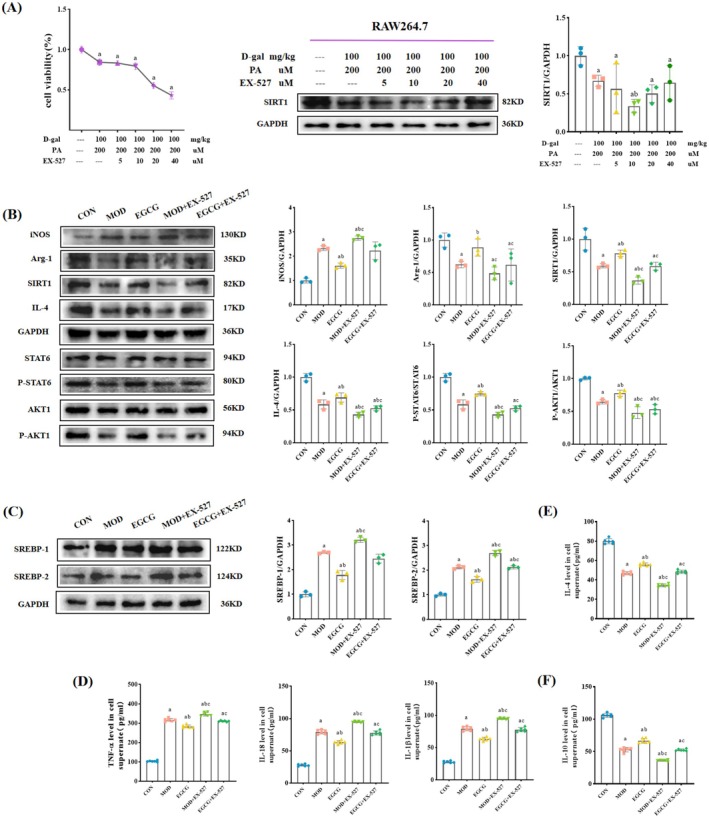
SIRT1 inhibitor EX‐527 increased M1‐like macrophage and lipid accumulation by EGCG in the model cells. (A) Determination of the optimal concentration of EX‐527. *n* = 3. (B) Representative WB images and quantitative analysis of the level of iNOS, Arg‐1, SIRT1, IL‐4, P‐STAT6, and P‐AKT1 in the RAW264.7 cells. *n* = 3 (C) Representative WB images and quantitative analysis of the level of SREBP‐1 and SREBP‐2 in the MPC5 cells. *n* = 3. (D) Levels of TNF‐α, IL‐18, and IL‐1β in supernatant. *n* = 6. (E, F) Levels of IL‐4 and IL‐10 in supernatant. *n* = 6. Compared with the CON group, ^a^ *p* < 0.05; compared with the MOD group, ^b^ *p* < 0.05; compared with the EGCG group, ^c^ *p* < 0.05.

**FIGURE 9:**
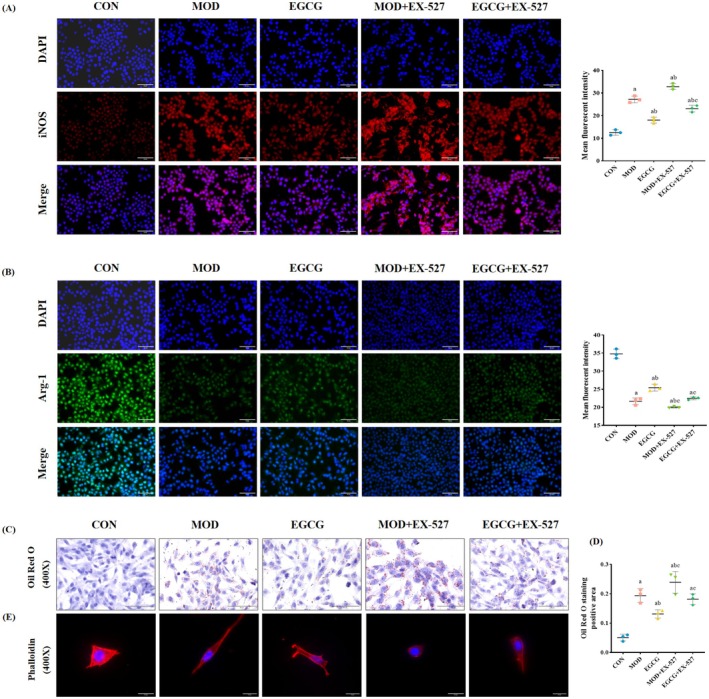
SIRT1 inhibitor EX‐527 increased M1‐like macrophage and lipid accumulation by EGCG in the model cells. (A) Representative iNOS immunofluorescence images (red fluorescence), quantitative analysis of the mean fluorescent intensity in each group of RAW264.7 cells. 400×, *n* = 3. (B) Representative Arg‐1 immunofluorescence images (green fluorescence), quantitative analysis of the mean fluorescent intensity in each group of RAW264.7 cells. 400×, *n* = 3. (C, D) Representative Oil Red O images of MPC5 cells and quantitative analysis of positive area. 400×, *n* = 3. (E) Representative Phalloidin staining images (red fluorescence) in MPC5 cells. 400×, *n* = 3. Compared with the CON group, ^a^ *p* < 0.05; compared with the MOD group, ^b^ *p* < 0.05; compared with the EGCG group, ^c^ *p* < 0.05.

EX‐527 was selected as an inhibitor to pretreat RAW264.7 cells to explore the mechanism of macrophage polarization and further observe the lipid accumulation of MPC5 cells. The results of CCK8 and WB (Figure 8A) showed that the optimal concentration of EX‐527 was 10 μM. Subsequently, SIRT1/AKT pathway proteins were detected to determine whether EX‐527 increased M1‐like macrophages. Figure 8B shows that, compared to the MOD group and the EGCG group, EX‐527 pretreatment increased the protein expression level of iNOS, decreased the protein expression levels of Arg‐1, SIRT1, IL‐4, and the phosphorylation levels of STAT6 and AKT1. At the same time, the treated RAW264.7 cells were immunofluorescently stained to observe if macrophage polarization existed. The results showed that compared with the MOD and EGCG groups, the mean fluorescence density of iNOS in the EX‐527 pretreatment group was increased, while Arg‐1 decreased (Figure 9A,B). This indicated that the SIRT1 inhibitor EX‐527 increased M1‐like macrophages.

Co‐culture treated macrophages with podocytes was to determine whether macrophage polarization affects podocyte lipid accumulation. First, inflammatory factors were detected. As expected, after EX‐527 pretreatment, levels of TNF‐α, IL‐18, and IL‐1β in supernatant increased, while levels of IL‐4 and IL‐10 in supernatant decreased (Figure 8D–F). Then, podocyte Oil Red O staining results showed an increase in the positive area of EX‐527 pretreatment compared to the MOD group and the EGCG group (Figure 9C,D). Phalloidin staining found that podocyte lipid accumulation was proportional to injury (Figure 9E). Further detection of the protein expression levels of SREBP‐1 and SREBP‐2 showed that the expression levels of the EX‐527 pretreatment group increased (Figure 8C). These suggested that the increase of M1‐like macrophages aggravates podocyte lipid accumulation.

### 3.8 SIRT1 Agonist SRT1720 Increased M2‐Like Macrophage and Decreased Lipid Deposition by EGCG in the Model Cells

**FIGURE 10:**
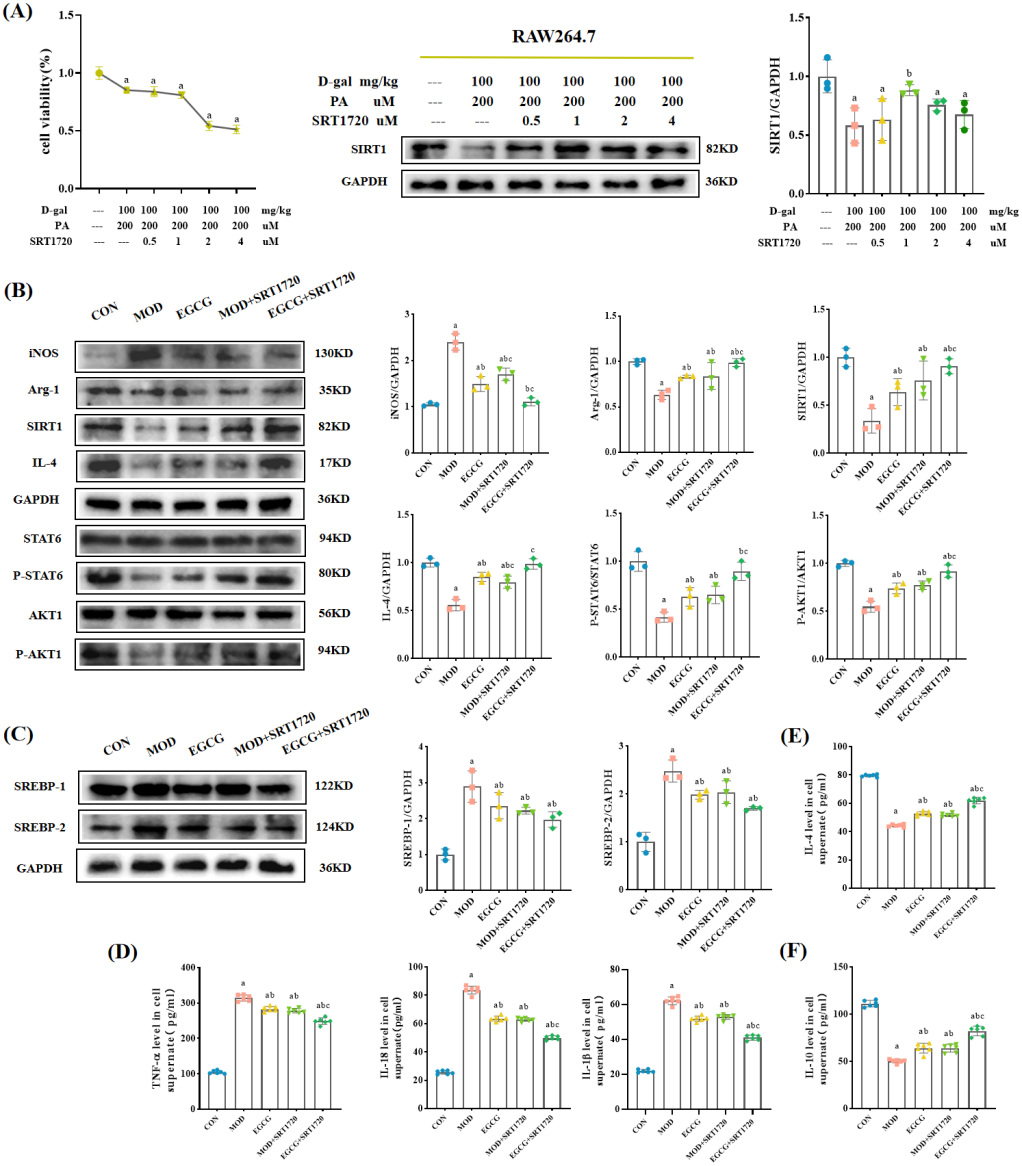
SIRT1 agonist SRT1720 increased M2‐like macrophage and decreased lipid deposition by EGCG in the model cells. (A) Determination of the optimal concentration of SRT1720 (*n* = 3). (B) Representative WB images and quantitative analysis of the level of iNOS, Arg‐1, SIRT1, IL‐4, P‐STAT6, and P‐AKT1 in the RAW264.7 cells (*n* = 3). (C) Representative WB images and quantitative analysis of the level of SREBP‐1 and SREBP‐2 in the MPC5 cells (*n* = 3). (D) Levels of TNF‐α, IL‐18, and IL‐1β in supernatant (*n* = 6). (E, F) Levels of IL‐4 and IL‐10 in supernatant (*n* = 6). Compared with the CON group, ^a^ *p* < 0.05; compared with the MOD group, ^b^ *p* < 0.05; compared with the EGCG group, ^c^ *p* < 0.05.

**FIGURE 11:**
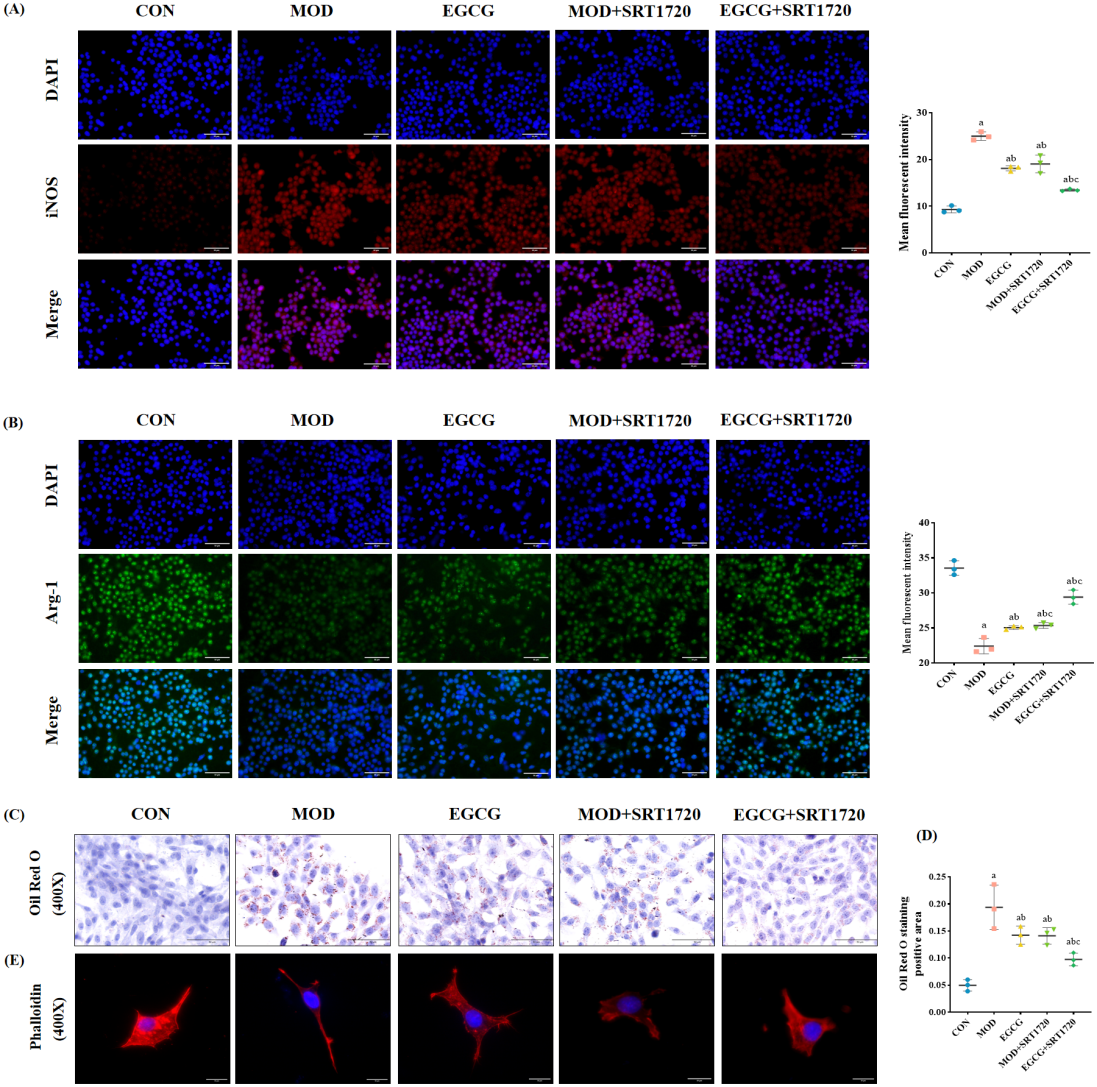
SIRT1 agonist SRT1720 increased M2‐like macrophage and decreased lipid deposition by EGCG in the model cells. (A) Representative iNOS immunofluorescence images (red fluorescence), quantitative analysis of the mean fluorescent intensity in each group of RAW264.7 cells. 400 ×, *n* = 3. (B) Representative Arg‐1 immunofluorescence images (green fluorescence), quantitative analysis of the mean fluorescent intensity in each group of RAW264.7 cells (400×, *n* = 3). (C, D) Representative Oil Red O images of MPC5 cells and quantitative analysis of positive area. 400×, *n* = 3). (E) Representative Phalloidin staining images (red fluorescence) in MPC5 cells. 400×, *n* = 3). Compared with the CON group, ^a^ *p* < 0.05; compared with the MOD group, ^b^ *p* < 0.05; compared with the EGCG group, ^c^ *p* < 0.05.

Similarly, based on the results of CCK8 and WB (Figure 10A), the concentration of SRT1720 pretreatment was selected as 1 μM. In contrast to EX‐527, the expression of iNOS decreased with SRT1720 pretreatment, and the expression of Arg‐1, SIRT1, IL‐4, and phosphorylation levels of STAT6 and AKT increased compared with the MOD group and the EGCG group. Immunofluorescence results further showed that SRT1720 pretreatment upregulated the mean fluorescence density of Arg‐1 and down‐regulated the mean fluorescence density of iNOS (Figure 11A,B).

Next, we measured the level of inflammation in each group of cells. Figure 10D–F shows that SRT1720 pretreatment enhanced the expression of anti‐inflammatory factors (IL‐4 and IL‐10) but weakened the expression of pro‐inflammatory factors (TNF‐α, IL‐18, and IL‐1β). Moreover, the results of Oil Red O and Phalloidin staining determined that SRT1720 pretreatment alleviated podocyte lipid accumulation and improved podocyte injury (Figure 11C–E). The WB results also showed that the expression levels of SREBP‐1 and SREBP‐2 proteins were reduced after SRT1720 treatment compared with the MOD group and the EGCG group (Figure 10C). These results prove that the SIRT1 agonist SRT1720 increased M2‐like macrophage levels and decreased lipid deposition by EGCG treatment in the model cells.

## 4 Discussion

Diabetic kidney disease (DKD), the most serious microvascular complication of DM, has become a critical disease endangering the health of the elderly (Jiang et al. 2020). Therefore, approaches for identifying effective and low‐toxicity natural botanical ingredients to promote aging with DKD are urgently needed. TP and EGCG are gradually attracting attention due to properties such as anti‐aging, treating hyperlipidemia, and anti‐inflammatory effects (Zhang et al. 2022). In terms of the properties, TP and EGCG have been confirmed to significantly prolong life by inducing mitochondrial autophagy (Sarah et al. 2023) and reduce the serum levels of TC and TG in hyperlipidemic rats (Wen et al. 2023). Other studies report that EGCG significantly ameliorates DKD by inhibiting the activation of the NLRP3 inflammasome (Zhang et al. 2024). Previous studies have mostly focused on the treatment of DKD without considering the effect of age. We thus analyzed the therapeutic effect of TP and EGCG on aging with DKD and explored the underlying mechanisms by in vivo and in vitro experiments. We found that TP mitigated aging with DKD by alleviating podocyte lipid accumulation, inflammation, and macrophage phenotypic imbalance.

A suitable model for the study of the pathogenesis and prevention of aging with DKD is especially important. D‐gal has been widely used to accelerate aging by generating reactive oxygen species (ROS) through converting to aldose and hydrogen peroxide under the catalysis of galactose oxidase (Azman and Zakaria 2019). Moreover, the activity of β‐galactosidase (SA‐β‐gal) and the expression level of P53 and P21 were significantly increased with D‐gal‐induced in our study (Suryadevara et al. 2024). Our previous research indicated that STZ injection combined with feeding a high‐glucose‐fat diet can selectively destroy pancreatic β cells, eventually leading to pancreatic injury and insulin resistance (Feng et al. 2024). Moreover, we also found a significant increase in Scr, BUN, 24‐h UTP, UACR, and the ratio of renal vacuolization under the administration of feeding a high‐glucose‐fat diet and injecting D‐gal and STZ. In addition, we report that the rats with kidney damage were accompanied by enhanced positive area of Oil Red O staining, serum TG, TC, LDL, and decreased serum HDL. Therefore, the rat model of aging with DKD possessing the features for lipid accumulation was successfully established. And TP intervention improved the lipid accumulation in the rat model.

Podocyte, the key part of the glomerular filtration barrier, is responsible for the major physiological and metabolic functions of the kidney. The injury to the podocyte is considered to drive the progression of both aging and DKD (Reidy et al. 2014; Shankland et al. 2021). A growing body of evidence shows that podocyte is particularly sensitive to lipid accumulation, which causes podocyte damage (Fu et al. 2020; Qu et al. 2024). Similarly, lipid accumulation in podocytes was observed in terms of the increased mean fluorescence density of adipophlin/SYNPO through immunofluorescence co‐staining in the MOD group. Meanwhile, we also found the thickened basement membrane, the fusion of foot processes, and the irregular arrangement of podocytes with the enlargement and disappearance by TEM. In this study, we suggested that EGCG treatment reduced podocyte damage by lipid accumulation induced by PA combined with D‐gal.

A hallmark pathogenesis for aging with DKD is the existence of persistent inflammation in kidney (Reidy et al. 2014; Shankland et al. 2021). The regulation of inflammatory processes in kidney is a matter of great interest for current researchers (Luo, Chen, et al. 2024; Wu et al. 2021). A previous study has reported that the onset and resolution of inflammation are mutually regulated by macrophage responses (Lu et al. 2023). Under the stimulation of inflammation, macrophages are recruited and release a series of proinflammatory factors, which aggravate organic inflammation. Notably, the result of single‐cell sequencing has revealed macrophage infiltration in the setting of the DKD environment, with macrophages expressing the more M1 phenotype marker (Fu et al. 2019; Shao et al. 2024). It is well known that M1‐like macrophages, accelerating the occurrence and development of inflammation, can secrete IL‐1β, IL‐18, TNF‐α and highly express iNOS; while M2‐like macrophages promote tissue repair by secreting IL‐10, IL‐4, and Arg‐1 (Cantero‐Navarro et al. 2021; Lin et al. 2024). Our study demonstrated that the levels of serum and kidney inflammation‐associated factors, and the expression of M1 phenotype macrophages were significantly increased, whereas rescued by continuous TP treatment.

Silent information regulator 1 (SIRT1), a putative regulator of biological longevity, gradually shows significance in the physiological functions of the kidney, including maintaining the homeostasis of cell differentiation, glycolipid metabolism, and inflammation (Perico et al. 2024; Wu et al. 2022). Previous studies have found that SIRT1 expression is significantly reduced in DKD, and activation of SIRT1 can protect renal function (Jin et al. 2024; Liu et al. 2022). Our study identified the activation of TP and EGCG on SIRT1 by detecting the expression level and molecular docking. Reportedly, SIRT1 regulates macrophage polarization to attenuate calcium oxalate‐induced kidney injury (Song et al. 2023). In our research, we also found that SIRT1 played a key role in macrophage polarization, and the imbalance of macrophage phenotype was improved by TP treatment.

The mechanism of macrophage polarization is complex and unclear, greatly attracting current researchers. We pretreated RAW264.7 cells with an inhibitor of EX‐27 and an agonist SRT1720 to investigate the mechanism of SIRT1 modulating macrophage polarization. STAT6 and AKT1 are downstream enzymes of SIRT1 and particularly active in macrophage polarization (Liu et al. 2019). AKT1, belonging to the AGC kinase family of serine/threonine kinases, regulates the cell cycle, transcription, translation, apoptosis, and differentiation; AKT1 phosphorylation contributes to macrophage differentiation into the M2 phenotype (Arranz et al. 2012). It has been verified that SIRT1‐mediated deacetylation is required for AKT1 phosphorylation in macrophages (Jia et al. 2023). In addition, activation of phosphorylated STAT6 is a key event in the transition of macrophages to an anti‐inflammatory, and silencing STAT6 can decrease IL‐4‐induced secretion of anti‐inflammatory factors (Kamerkar et al. 2022; Liu et al. 2017). A previous study verified that SIRT1 enhances STAT6‐Tyr641 phosphorylation by inhibiting the expression of protein tyrosine phosphatase 1B (PTP1B) (Lu et al. 2008; Sun et al. 2007). Our experimental results also confirmed that inhibiting SIRT1 reduces the M2‐like macrophages and the phosphorylation level of AKT1 and STAT6 in RAW264.7 cells induced by PA combined with D‐gal. Conversely, overexpression of SIRT1 increases the M2‐like macrophages and the phosphorylation level of AKT1 and STAT6. Our data clarified that activation of P‐AKT1and P‐STAT6 signaling was required for the protective effect of TP in aging with DKD, targeting SIRT1‐mediated macrophage polarization.

Inflammation is known to have a feedback effect on lipid metabolism (Luo, Chen, et al. 2024; Luo, Luo, et al. 2024). Previous studies show that inhibition of the NLRP3 inflammasome could improve podocyte injury by alleviating lipid accumulation in DKD (Wu et al. 2021). However, the effect of macrophages on lipid accumulation in podocytes has not been reported. In our study, the co‐culture system of RAW264.7 and MPC5 cells was constructed to explore the role of SIRT1‐mediated macrophage polarization in podocyte lipid accumulation. Sterol regulatory element‐binding protein (SREBP) is a transcription factor dominating the expression of lipid synthesis‐related genes and is closely related to lipid droplet formation. SREBP‐1a is involved in overall lipid synthesis and growth, SREBP‐1c is responsible for fatty acid synthesis and energy storage, and SREBP‐2 is involved in cholesterol synthesis (Shen et al. 2024). The tight binding of phalloidin to F‐actin can reveal the distribution of the filament skeleton in the cell, which can be used to determine the degree of podocyte injury (Wu et al. 2021). Our results of phalloidin staining suggested that EGCG attenuated podocyte injury by alleviating lipid accumulation. Oil Red O staining analysis showed that EGCG intervention mitigated lipid accumulation in podocytes through modulating the SIRT1‐mediated macrophage polarization. Moreover, protein expression results demonstrated that EGCG decreased SREBP‐1 and SREBP‐2 after reducing M1 macrophages. All these results implied that TP treatment prevented podocyte lipid accumulation, at least in part, through regulating macrophage polarization.

## 5 Conclusion

In conclusion, our research indicated for the first time that TP and EGCG treatment is used for preventing podocyte lipid accumulation and damage in the model of aging with DKD through activation of SIRT1‐mediated macrophage polarization. This study not only suggests that SIRT1 is a powerful potential target for the prevention of DKD in the elderly, but also has an important significance based on targeted macrophage treatment of DKD.

## Author Contributions

**Shuangzhi Chen:** data curation (lead), funding acquisition (supporting), project administration (lead), writing – original draft (lead). **Xi Wang:** methodology (equal), project administration (equal). **Chengyang Li:** methodology (equal), project administration (equal). **Le Cheng:** methodology (equal), project administration (equal). **Chenhui Lv:** visualization (equal). **Lushan Xue:** project administration (equal). **Cheng Zhang:** project administration (equal). **Xuemin Li:** methodology (equal). **Mingkai Li:** software (equal). **Qinfei Guo:** software (equal). **Yafei Zhao:** software (equal). **Haifeng Zhao:** conceptualization (equal), data curation (supporting), funding acquisition (lead), writing – review and editing (lead).

## Ethics Statement

All animal experiments comply with the ARRIVE guidelines and are approved by the Institutional Animal Care and Use Committee of Shanxi Cancer Hospital (Issue No. 2022002).

## Conflicts of Interest

The authors declare no conflicts of interest.

## Data Availability

Data will be made available on request.
